# A New Specific and Sensitive RT-qPCR Method Based on Splinted 5′ Ligation for the Quantitative Detection of RNA Species Shorter than microRNAs

**DOI:** 10.3390/ncrna7030059

**Published:** 2021-09-18

**Authors:** Marine Lambert, Abderrahim Benmoussa, Patrick Provost

**Affiliations:** 1 CHU de Québec Research Center/CHUL Pavilion—Université Laval, 2705 Blvd Laurier, Quebec City, QC G1V 4G2, Canada; marine.lambert@mail.mcgill.ca (M.L.); abderrahim.benmoussa@umontreal.ca (A.B.); 2 Department of Microbiology, Infectious Diseases and Immunology, Faculty of Medicine, Université Laval, Quebec City, QC G1V 0A6, Canada

**Keywords:** small RNA, 5′ ligation, RT-qPCR, splint, adapter, quantification, methods for functional RNA studies

## Abstract

Recently, we discovered a new family of unusually short RNAs mapping to 5.8S ribosomal RNA (rRNA) and which we named dodecaRNAs (doRNAs), according to the number of core nucleotides (12 nt) their members contain. To confirm these small RNA-sequencing (RNA-Seq) data, validate the existence of the two overly abundant doRNAs—the minimal core 12-nt doRNA sequence and its + 1-nt variant bearing a 5′ Cytosine, C-doRNA—and streamline their analysis, we developed a new specific and sensitive splinted 5′ ligation reverse transcription (RT)-quantitative polymerase chain reaction (qPCR) method. This method is based on a splint-assisted ligation of an adapter to the 5′ end of doRNAs, followed by RT-qPCR amplification and quantitation. Our optimized protocol, which may discriminate between doRNA, C-doRNA, mutated and precursor sequences, can accurately detect as low as 240 copies and is quantitatively linear over a range of 7 logs. This method provides a unique tool to expand and facilitate studies exploring the molecular and cellular biology of RNA species shorter than microRNAs.

## 1. Introduction

Small non-coding RNAs are a class of non-coding RNAs that are shorter than 200 nucleotides (nt). In recent years, small RNAs have been characterized as key regulators of several fundamental transcriptional and post-transcriptional cellular mechanisms [1,2,3]. Next-generation sequencing (NGS) technologies have greatly contributed to the discovery and study of these small RNAs, especially through the nucleotide sequence length and composition that they provide, allowing to work without any prior sequence knowledge. 

To monitor and analyze the expression of known small RNAs, however, a microarray and reverse transcription (RT)-quantitative polymerase chain reaction (qPCR) become the methods of choice. Whereas microarray approaches allow for large-scale expression profiling studies of known sequences, RT-qPCR quantitation remains the most widely used, relatively simple, straightforward and least expensive method to routinely monitor and quantitate the levels of known small RNAs of interest in research laboratories. The RT-qPCR quantitate expression of specific RNA sequences by exponential amplification mostly uses a fluorescent reporter, either via a DNA intercalating agent (SYBR green) [4,5] or using fluorescent probes complementary to the targeted RNA [6]. We can, thus, quantitatively detect 19 to 24-nt microRNAs, even from low-input samples.

There are two types of quantitation based on RT-qPCR: while relative quantification lies on the ratio of target RNA found in unknow samples compared to a control sample, absolute quantitation consists of calculating the number of RNA copies using a standard curve established with known amounts of the synthetic form of the RNA of interest. In every case, quantification can be normalized and improved by addition to the samples of a synthetic small RNA sequence absent from the studied cells, tissues or species (known as a spike-in) [7], or using an endogenous small RNA, which has a constant expression in both tested and control samples (e.g., small nuclear RNA U6) [8,9].

Over the recent years, the use of NGS, microarray and RT-qPCR provided the research community with the tools that allowed researchers to improve their grasp upon the diverse classes of small RNAs and allowed a better understanding of their function. This is exemplified by an increasing number of studies demonstrating the functional role and biological significance of non-coding RNA fragments, such as transfer RNA (tRNA)-related RNA fragments (tRFs) [10] or rRNA-related RNA fragments (rRFs) [11,12], while these fragments were, until recently, considered as mere products of degradation. The role of tRFs in the translation regulation mechanisms, especially during cellular stress, has become widely recognized [13,14]. Less is known about rRFs, although a recent study has demonstrated their role in translation regulation and embryogenesis [15]. Moreover, recent studies from our laboratory have shown that some small RNAs shorter than 16 nt, derived either from microRNAs [16] or ribosomal RNA (rRNA) [17], may exist and function within cells.

There is, currently, no method available to specifically detect and quantitate known small RNAs shorter than 16 nt. If it were to exist, such a method would greatly facilitate the study of unusually short RNAs, accelerate the pace of research in that field in both prokaryotes and eukaryotes, improve our understanding of their biology and anticipate their possible use as biomarkers or in therapeutics. Such a method would need to be designed to detect and quantitate the levels of specific RNA sequences in a specific and sensitive manner, even if or when these small RNAs derive from abundant and longer RNA precursor sequences (e.g., rRNA, tRNA, microRNA).

Recently, a small RNA-Seq analysis of 11 samples derived from six different species revealed a new family of RNAs shorter than microRNAs. The two main sequences found were one of 12 nt, which we named dodecaRNA (doRNA), and its 13-nt variant, which differs only by an additional Cytosine at the 5′ end and was named C-doRNA [17]. These two sequences were found in all the mouse and human samples that we analyzed. Here, we report the development of a new splinted 5′ ligation RT-qPCR method aimed to validate small RNA-Seq data and confirm the existence of doRNA and C-doRNA sequences in eukaryotes. 

## 2. Results

This section may be divided by subheadings. It should provide a concise and precise description of the experimental results, their interpretation, as well as the experimental conclusions that can be drawn.

### 2.1. Detection of doRNAs by a New Splinted 5′ Ligation RT-qPCR Method

The short doRNA (12 nt) and C-doRNA (13 nt) are the two most abundant sequences of the family of dodecaRNAs (doRNAs) that we discovered recently in human and mouse small RNA transcriptomes (please refer to the manuscript submitted as a separate Standard Paper) [17]. The only difference between these two sequences is the extra Cytosine (C) present at the 5′ end of C-doRNA. In addition, our results suggested that doRNA and C-doRNA may be generated upon the processing of longer rRNA precursors containing 5.8S rRNA [17], thereby posing a major challenge to their specific detection, among abundant precursors containing the same sequences, and quantitation, 19 to 24-nt microRNA species being the shortest RNAs that can be amplified and quantitated by qPCR.

**Figure 1:**
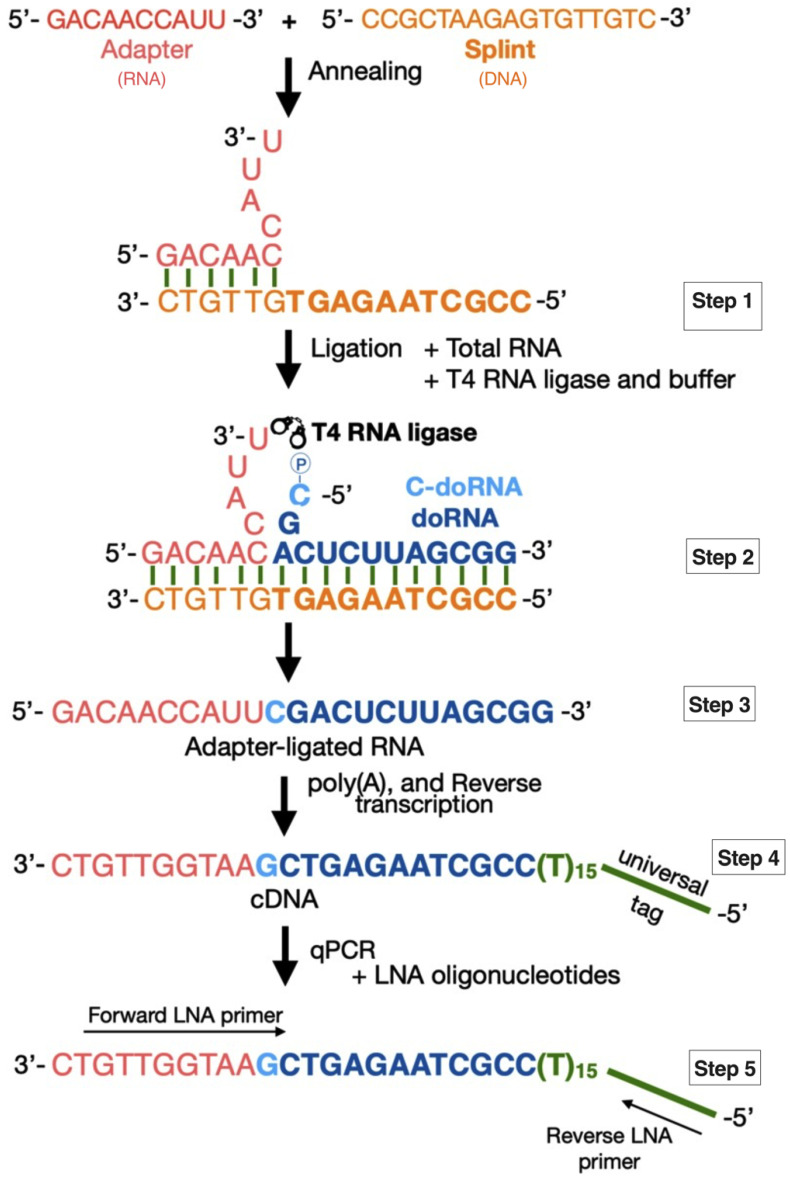
Schematic representation of the splinted 5′ ligation RT-qPCR method established to detect doRNAs. In Step 1, the 5′ RNA adapter was pre-annealed with the 4:2 splint DNA. In Step 2, the 5′ adapter:splint complex was added to total RNA to target the adapter ligation to the doRNA and the C-doRNA by base pairing; the splint specified the adapter ligation to the 5′ end of the doRNA or C-doRNA sequence. The ligation of the adapter to the doRNA or C-doRNA, templated by the splint, required the 5′ phosphate (marked as an encircled P) of the doRNA or C-doRNA, and was mediated by the T4 RNA ligase. The resulting adapter-ligated RNA intermediate (Step 3) was reverse transcribed by adding a poly(A) tail, and cDNA was synthesized using a poly(T) primer with a 3′ degenerate anchor and a 5′ universal tag (Step 4). Finally, doRNA and C-doRNA, 5′ extended by the adapter, were quantitated by qPCR using a set of locked nucleic acid (LNA)-modified oligonucleotides, with the forward primer specific to the RNA, and the reverse primer specific to the universal tag (Step 5).

In order to take up this technical challenge, we set-up a new detection method, based on RT-qPCR, but preceded by a 5′ adapter ligation step aimed at (i) lengthening doRNA and C-doRNA to the size of a microRNA, and (ii) positioning the discriminating 5′ C at the center of the adapter-ligated sequences (Figure 1). In this 5′ adapter ligation step, we made use of a single-stranded splint sequence annealed, through 6 of its 17 nt, to a 10-nt RNA adapter, leaving a single-stranded extension of 11 nt designed to hybridize with most of the doRNA or C-doRNA sequence. The splint-assisted ligation of the adapter extended doRNA and C-doRNA into new RNA sequences of 22 nt and 23 nt that did not map to human and mouse transcriptomes (see Materials and Methods Section) and that could then be conventionally amplified and quantitated by qPCR.

After ligation, the adapter-ligated RNA products were polyadenylated to introduce a poly(A) tail at the 3′ end, followed by reverse transcription using a poly (dT) oligonucleotide containing a Universal primer (UP) in its floating tail, and a 3′ degenerated anchor. This step was crucial, since it differentiated our RNAs of interest from their precursors elongated on their 3′ side, which complemented the splint-mediated ligation of the adapter that specified and discriminated their 5′ side.

The final step consisted of the qPCR quantitation of our RNAs of interest using LNA oligonucleotides [5,18,19]. These LNA primer sets had their ribose ring “locked” in the ideal conformation for the Watson–Crick base pairing and allowed for a higher thermal stability when hybridized to a complementary DNA or RNA strand. This feature conferred a markedly improved specificity to these primers, which allowed us to distinguish RNAs that differed by a single nt at their 5′ end and their 3′ end.

### 2.2. Optimization of the Ligation Reaction

The efficiency of doRNA and C-doRNA-splinted 5′ ligation of an adapter was a major determinant of the specificity and sensitivity of our detection method. In order to optimize the splinted 5′ ligation step, we optimized the buffer composition and the sequence of the splint. First, the adapter was always added in excess during ligation, in order to avoid adapter dimers while ensuring a high ligation efficiency. The adapter:target RNA ratio gave equivalent ligation efficiencies; however, ligation using the ratio of ~five adapters to one target gave slightly better results (Supplementary Figure S1, fold change between 1.126 and 1.136 for doRNA and C-doRNA detection, respectively). 

To catalyze the ligation reaction, we used T4 RNA ligase, an enzyme that requires a free 5′ phosphate on the donor RNA (doRNA or C-doRNA), a 3′ hydroxyl on the acceptor RNA (adapter RNA) and ATP [20]. The synthetic RNA adapter was devoid of 5′ phosphate, so to prevent them from being ligated to each other. Moreover, in order to specify the ligation of our adapter to the doRNA and C-doRNA sequences, we opted for the use of a DNA splint [21]. However, the T4 RNA ligase is preferentially active when the nucleotides at the junction are single-stranded [21]. Thus, the DNA splint was designed so to bring the donor and acceptor RNAs close to each other, while leaving single stranded regions near the ligation junction. Since the optimal length of each single-stranded region at the junction varies according to the sequences [21], we tested different lengths. We named the splints that we used according to the number of free nucleotides in the adapter (the first number), and the number of free nucleotides in the C-doRNA-ligated part (the second number). Both numbers were separated by “:”. For instance, the splint 4:2 was designed to leave 4 nt free from the adapter RNA and 2 nt from the C-doRNA (or only one free nt for the doRNA; please refer to Step 2 of Figure 1, dark vs. light blue).

**Figure 2:**
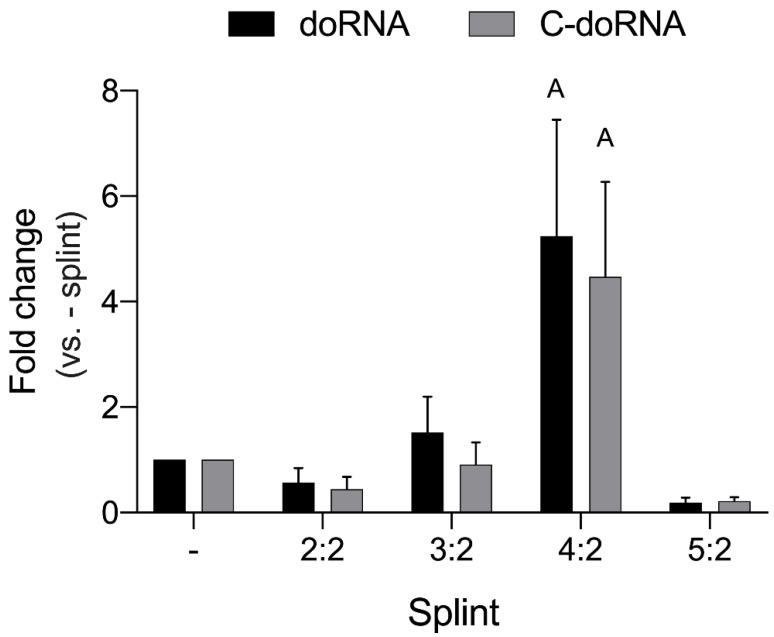
Optimization of the DNA splints. Splints of various length were assessed for their ability to optimize 5′ ligation RT-qPCR detection of doRNA and C-doRNA. Cycle quantitation (Cq) data were normalized on U6 RNA, and the fold changes were calculated versus the optimal 4:2 splint condition (mean ± SEM; *n* = 3 independent experiments). Letters above the bars represent conditions with a normalized fold change significantly different from the control (without splint,-splint): A, *p* < 0.0097 (two-way ANOVA with Holm–Sidak’s post hoc test).

Our assays suggested that the splint yielding the most efficient ligation was the 4:2 splint for doRNA and for C-doRNA (Figure 2, 4:2 splint: fold change between 5.2 and 4.5). We also found the ratio of one DNA splint to two RNA adapters to be the optimal one (Supplementary Figure S2).

**Figure 3:**
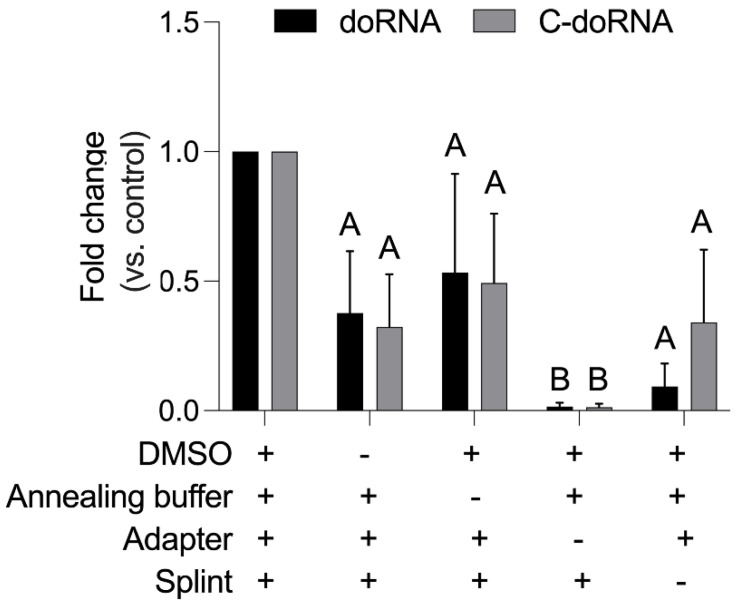
Assessment of the importance of the main reaction medium components. The importance of the essential components of the splinted 5′ ligation buffer was assessed by quantitatively evaluating doRNA and C-doRNA detection in their absence (−) or presence (+). Cycle quantitation (Cq) data were normalized on U6 RNA, and the fold changes were calculated versus the optimized buffer condition (control) containing all the components (mean ± SEM; *n* = 3 independent experiments). The 4:2 splint was used in this experiment. Letters above the bars represent conditions with a normalized fold change significantly different from the control: A, *p* < 0.0260; B, *p* < 0.0399 (two-way ANOVA with Holm–Sidak’s post hoc test).

In order to identify the most efficient workflow for the doRNA and C-doRNA-splinted 5′ ligation RT-qPCR, we analyzed the Cq results from qPCR obtained from analyzing total RNA extracts of mouse neuronal cells, the N2a. These were used because of their high levels of doRNAs. We, first, determined that pre-annealing the splint with the adapter, before adding them to the reaction, improved doRNA and C-doRNA ligation (Supplementary Figure S3, fold change between 188 and 51 for doRNA and C-doRNA detection, respectively). Moreover, we found that the addition of an annealing buffer containing salts (200 mM Tris-HCl, 750 mM KCl) could improve the stability and hybridization of our RNAs and, consequently, the efficiency of ligation (Figure 3; Annealing buffer, fold change between 0.53 and 0.49).

We assessed the importance of each component of the splinted 5′ ligation buffer by quantitatively evaluating the level of doRNA and C-doRNA detection in their absence. These assays confirmed that the splinted 5′ ligation RT-qPCR detection of doRNA and C-doRNA, in total RNA extracted from mouse neuronal N2a cells, was optimal in the presence of the following eight (8) components that were tested: DMSO, PEG8000, ATP, T4 RNA ligase buffer, RNase inhibitor, annealing buffer, adapter and splint (Figure 3, Supplementary Figure S4). DMSO is often used to troubleshoot ligation reactions that may be compromised by secondary RNA structures shielding the extremities [22,23,24], and it seems that 10% (*v*/*v*) DMSO may also optimize our splinted 5′ ligation RT-qPCR detection method by improving the efficiency of the 5′ ligation step (Figure 3; DMSO, fold change between 0.37 and 0.32). 

We supplemented the ligation reaction mixture with 25% PEG8000, which is known to enhance intermolecular ligation by increasing the concentration of donor and acceptor ends by macromolecular crowding [25]. Indeed, we observed a dramatic >95% reduction in doRNA or C-doRNA detection upon the removal of PEG8000, which attested of its utility (Supplementary Figure S4; PEG8000, fold change between 0.040 and 0.023). T4 RNA ligase uses ATP to catalyze the linkage of two RNA molecules [20]. Therefore, it did not come as a surprise that its omission markedly reduced the splinted 5′ ligation RT-qPCR detection of doRNA and C-doRNA by >90% (Supplementary Figure S4; ATP, fold change between 0.075 and 0.046). Even with the addition of ATP, the ligation buffer for the T4 RNA ligase (50 mM Tris-HCl, 2 mM MgCl_2_, 1 mM DTT pH 7.5) proved to be essential to the reaction (Supplementary Figure S4; T4 RNA ligase buffer, fold change between 0.022 and 0.001).We could see that the RNase inhibitor, which was expected to preserve RNAs during the ligation step, improved the detection of doRNA and C-doRNA by at least three-fold (Supplementary Figure S4; RNase inhibitor, fold change between 0.26 and 0.09), whereas the annealing buffer improved the detection signal at most by two-fold.

Notably, while the doRNA and C-doRNA detection levels were >85% lower in the absence of the splint (Figure 3; splint, fold change between 0.10 and 0.15), the reduction in doRNA and C-doRNA detection reached 99% in the absence of adapter RNA (Figure 3; adapter, fold change between 0.011 and 0.010), used here as a negative control, confirming their relative importance to the sensitivity of our assay.

### 2.3. Splinted 5′ Ligation RT-qPCR Specifically Amplify doRNA and C-doRNA

**Figure 4:**
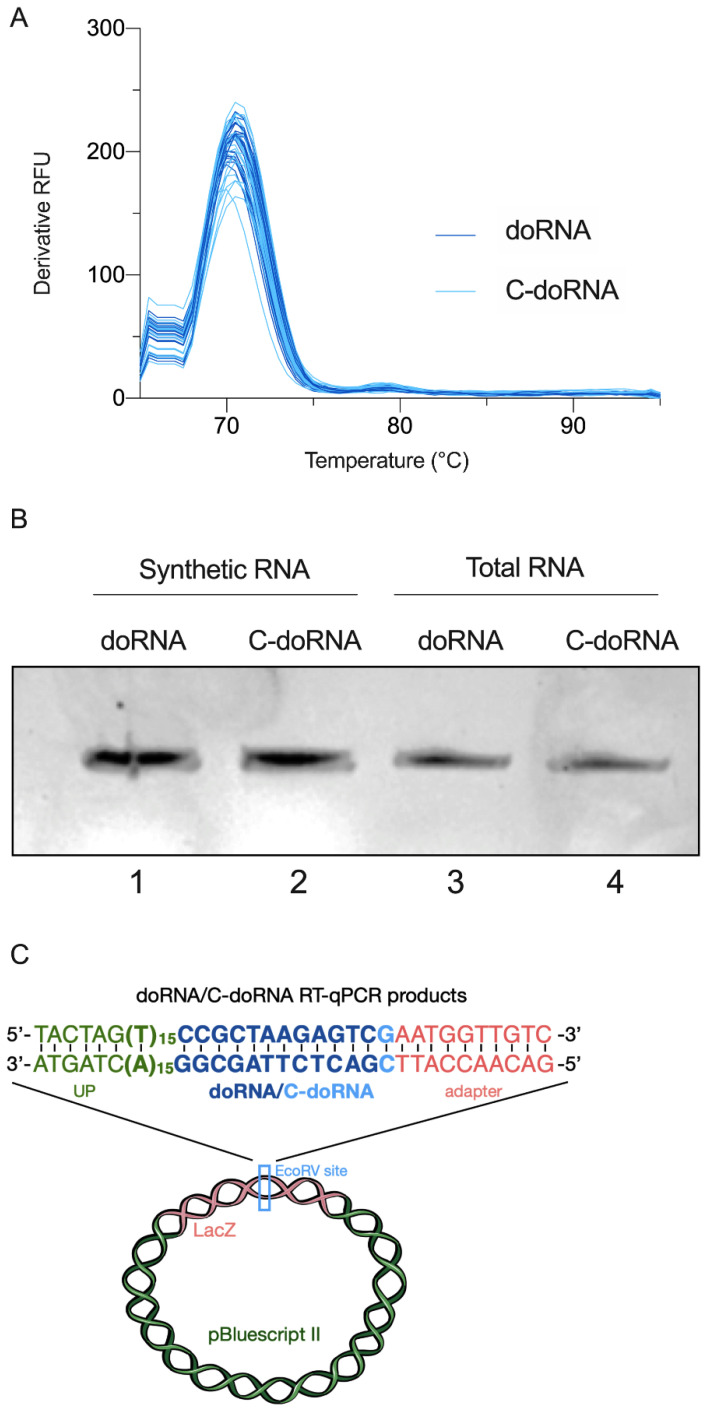
Amplification of doRNA and C-doRNA by splinted 5′ ligation RT-qPCR was specific. (**A**) Melting curve of the qPCR reaction products following splinted 5′ ligation RT-qPCR detection of doRNA (in dark blue) or C-doRNA (in light blue) in total RNA samples from cultured N2a cells. RFU, relative fluorescence unit. (**B**) Amplification of a single product upon splinted 5′ ligation RT-qPCR detection of RNA and C-doRNA. qPCR products were separated by 20% acrylamide/8 M urea gel electrophoresis and visualized by nucleic acid staining (RedSafe). (**C**) DNA contained in the bands excised from lanes 3 and 4 was extracted and cloned into the pBluescript II plasmid. DNA sequencing confirmed the identity of the expected amplified product (in color). UP, universal primer.

The melting curve performed after the splinted 5′ ligation RT-qPCR amplification of doRNA or C-doRNA products, from synthetic doRNA or C-doRNA or from total RNA extracted from N2a cells, showed a unique peak, suggesting that, in each case, a unique RNA product was amplified (Figure 4A). This was confirmed by 20% acrylamide/8 M urea gel electrophoresis, which revealed a single band (Figure 4B). Cloning and sequencing of the RT-qPCR cDNA products contained in these bands revealed the identity of the 42 and 43-nt sequences expected from 5′ adapter-ligated and 3′ poly(A)-oriented, universal primer-assisted doRNA and C-doRNA amplification, respectively (Figure 4C). We were, thus, able to confirm that our method allowed the specific amplification of doRNA and C-doRNA in a total RNA sample.

### 2.4. Splinted 5′ Ligation RT-qPCR May Discriminate between doRNA, C-doRNA and Precursor Sequences

**Figure 5:**
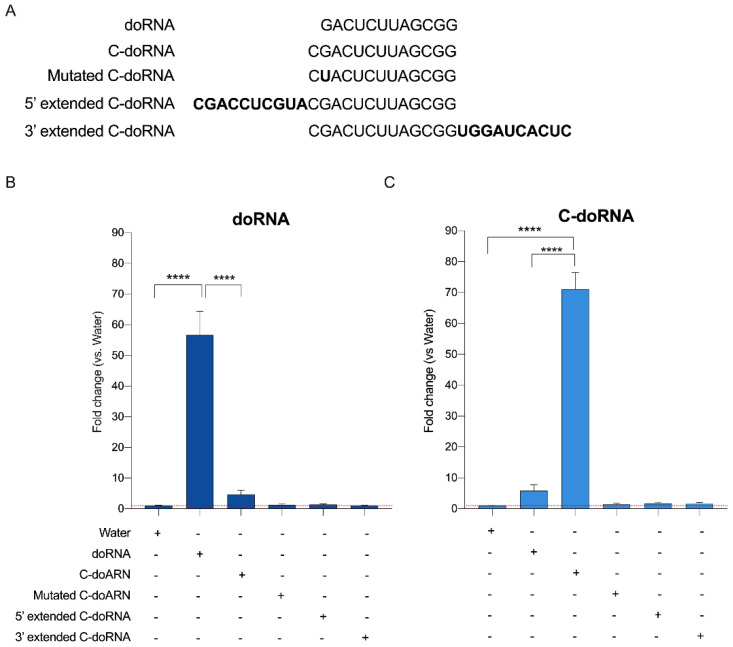
Splinted 5′ ligation RT-qPCR may discriminate between doRNA, C-doRNA and precursor sequences. (**A**) Synthetic oligonucleotide sequences used to test the specificity of our splinted 5′ ligation RT-qPCR method. Mutated C-doRNA was obtained by substituting G2 to U2 near the 5′ end. The 5′ and 3′ extended C-doRNAs were 10-nt extensions of C-doRNA in the 5′ or 3′ direction of the 5.8S rRNA precursor sequence, respectively. (**B**,**C**) Total RNA (300 ng) from N2a cells was used, followed by addition of 6.02 × 10^9^ copies of the indicated synthetic oligonucleotides (or water, as control) in a final volume of 2 µL. The qPCR data were normalized on U6 RNA levels, and the fold change was calculated by monitoring doRNA (**B**) or C-doRNA (**C**) versus the control sample (mean ± SEM; *n* = 4 independent experiments). The symbol (+) denotes the presence, and (−) the absence of the indicated component. The horizontal dotted red line indicates a fold change of 1 (no change). **** *p* < 0.0001 (one-way ANOVA with Holm–Sidak’s post hoc test).

To assess the specificity of doRNA and C-doRNA detection, we quantitated doRNA and C-doRNA levels in total RNA (300 ng) from N2a cells, which already contained doRNA and C-doRNA, supplemented either with water (control) or 6.02 × 10^7^ copies of synthetic oligonucleotides (doRNA, C-doRNA, mutated C-doRNA, 5′ extended C-doRNA or 3′ extended C-doRNA) (Figure 5A). C-doRNA was extended using sequences identical to those bordering it in rRNA precursors. We were able to demonstrate the relative specificity of the splinted 5′ ligation RT-qPCR method in detecting doRNA using the doRNA-LNA primer (Figure 5B, fold change of 56) and C-doRNA with the C-doRNA-LNA primer (Figure 5C, fold change of 71). We detected a low level of cross-detection that did not reach statistical significance, when doRNA detection increased upon C-doRNA addition (Figure 5B, fold change of 4.6) and, conversely, when C-doRNA detection increased upon doRNA addition (Figure 5C, fold change of 5.8). We observed no change in doRNA and C-doRNA detection when adding C-doRNA that bore a mutation at position two (G to U) or when adding 5′ or 3′ extended C-doRNAs, confirming that splinted 5′ ligation RT-qPCR may discriminate between doRNA, C-doRNA, mutated as well as precursor sequences. 

### 2.5. doRNA and C-doRNA Detection by Splinted 5′ Adapter RT-qPCR Is Linear over a Range of 7 Logs

**Figure 6:**
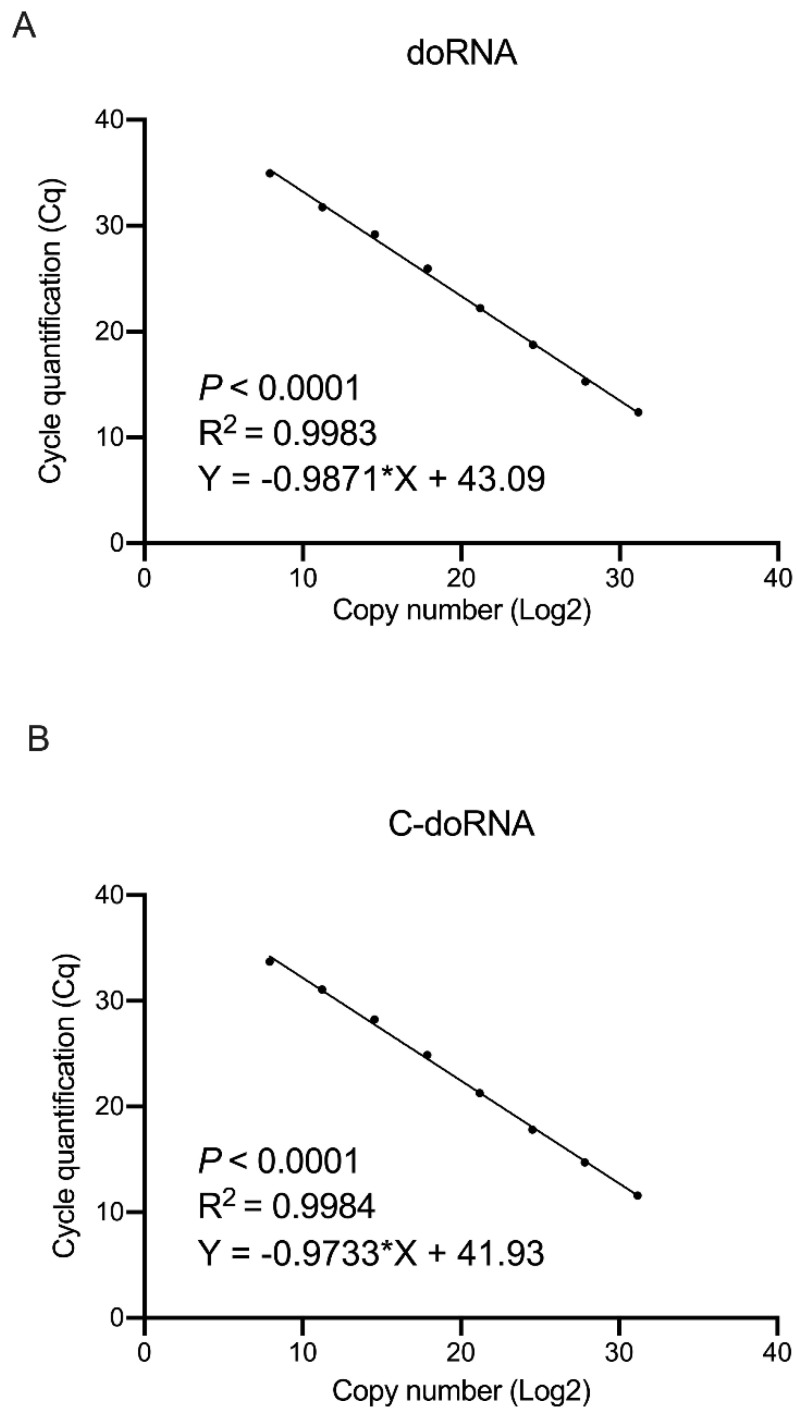
doRNA and C-doRNA detection by splinted 5′ adapter RT-qPCR is linear over a range of 7 logs. (**A**,**B**) Standard curves were established using synthetic doRNA (**A**) or C-doRNA (**B**) serial dilution to calculate doRNA or C-doRNA copy numbers in various cell and tissue samples.

To determine if our method could be used for the absolute quantitation of doRNA and C-doRNA in total RNA samples, we established standard curves using synthetic doRNA and C-doRNA, as well as miR-25 and miR-30a, from which the Cq results could be associated with their respective copy numbers. We observed that the doRNA and C-doRNA detection by splinted 5′ adapter RT-qPCR was sensitive and linear over a range of 7 logs (*R*^2^ > 0.9983, *p* < 0.0001), allowing their quantitation from a number of copies as low as 240 (Figure 6). This level of sensitivity was sufficient to quantitate doRNA and C-doRNA levels in the biological sample (human platelets) that contained the least doRNA (1235 copies) and C-doRNA (8520 copies).

### 2.6. Splinted 5′ Ligation RT-qPCR Validation of Small RNA-Seq Data

**Figure 7:**
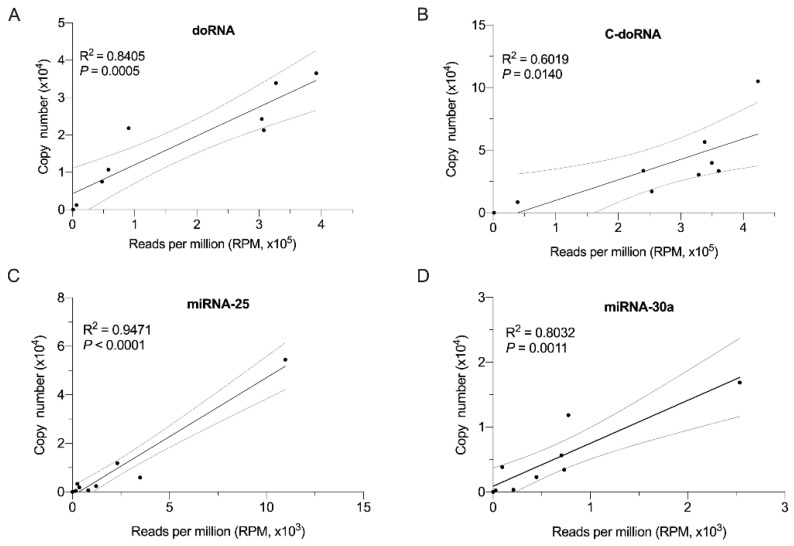
Splinted 5′ ligation RT-qPCR validation of small RNA-Seq data. Correlation between the relative abundance of doRNA (**A**), C-doRNA (**B**), miR-25 (**C**) and miR-30a (**D**) assessed by splinted 5′ ligation RT-qPCR or small RNA-Seq. qPCR data are represented as the average copy number from 3 technical replicates, while small RNA-Seq data are represented as the number of reads per million for one replicate. Linear models were used to plot the line of best fit for each correlation with confidence of 95%; the *R*^2^ value of the correlation is indicated.

We applied our splinted 5′ ligation RT-qPCR method to validate the small RNA-Seq profile of doRNA, C-doRNA, miR-25 and miR-30a in the same total RNA samples derived from eight (8) primary and cultured human and mouse cells and tissue that were analyzed. The difference between doRNA/C-doRNA and miR-25/miR-30a levels, when compared by small RNA-Seq, was less pronounced when assessed using our splinted 5′ ligation RT-qPCR method, which was expected to be more quantitative than small RNA-Seq. We observed a strong correlation between the levels of these four small RNA species, when the data expressed in terms of copy numbers versus RPM were plotted (Figure 7, *R*^2^ > 0.6019, *p* < 0.0140). The correlation was stronger for the 22-nt microRNAs than for the 12 and 13-nt doRNAs, which may be explained by their difference in length and, for microRNAs, by the detection of the authentic sequences without the need of adapter ligation.

## 3. Discussion

Very small RNAs are important regulators of gene expression which present unique challenges in quantification due to their size. Highly sensitive, straightforward (the ligation RT-qPCR protocol takes ~5 h to complete) and affordable methods are necessary to provide accurate and complete information on very small RNA profiles and functions. In this study, we designed a novel splinted 5′ ligation-based RT-qPCR that allowed the detection of a new family of doRNAs half the length of microRNAs. The crucial step of the protocol involved splint-mediated ligation of an RNA adapter, a highly efficient and low-bias approach that may improve ligation efficiency and specificity. High ligation efficiency is important to increase yield and allow for low input samples.

Adapter ligation to the 5′ end of small RNAs is particularly well described and used in small RNA library construction involved in small RNA-Seq. Several comparative studies on 3′ or 5′ ligation of total RNA exist [26,27,28], but, to the best of our knowledge, none have been developed for the ligation to a specific small RNA sequence. Only a few studies used a (randomized) DNA splint to increase the ligation efficiency of an adapter [29], but, again, these approaches were not designed for a specific small RNA. The use of T4 RNA ligase 1 was justified by the presence of a shorter splint that allowed the ligation of leaving single stranded regions (i.e., unpaired nucleotide) near the ligation junction. However, this method could be modified to include a splint perfectly complementary in all length of the adapter and the RNA of interest to use the T4 RNA ligase 2. Finally, the use of LNA-modified oligonucleotides in the qPCR amplification of doRNAs was instrumental in achieving the specificity of hybridization and amplification. 

The strong correlation of the microRNA data obtained by splinted 5′ ligation RT-qPCR versus small RNA-Seq suggests that our method allowed the detection of doRNAs without adding a bias in microRNA detection. Splinted 5′ ligation RT-qPCR also demonstrated its utility when assessing the differential expression of small RNAs between several different cell or tissue samples, which is commonly used in clinical diagnostic and more fundamental research. It may also enable the discovery and study of the gene targets or processes possibly regulated by these unusually short RNAs and provide clues on their role in cellular and molecular biology in health and diseases. 

The splinted 5′ ligation RT-qPCR protocol may be easily adapted and optimized to detect any RNA sequences of interest shorter than microRNAs, certainly down to 12 nt in length, as shown in this Method paper, and possibly even a little shorter, albeit expectedly with a variable specificity and sensitivity. The discovery of doRNAs, containing a core sequence of only 12 nt, may open a new area of research, and the splinted 5′ ligation RT-qPCR detection method that we set-up may be instrumental to achieve significant advances that would otherwise not be possible. 

We showed here that our method could detect two small RNAs shorter than 16 nt and did not interfere with the detection of two microRNAs using regular microRNA oligonucleotides. However, the use of our approach remains to be validated for other unusually short RNA sequences. The splinted 5′ ligation RT-qPCR method that we established, depicted in Figure 1, may be adapted to any sequences shorter than microRNAs and prove to be useful to researchers interested in this new area of non-coding RNA research. It is only by their independent use, assessment and validation that we will be able to evaluate the value, usefulness and limits of our proposed methodology.

This new method is relatively simple and straightforward to establish, perform routinely and scale-up in a molecular biology laboratory, and may help researchers explore and understand the biology and importance of RNA species shorter than microRNAs [11,30]. 

## 4. Materials and Methods

### 4.1. RNA and DNA Oligonucleotides

All synthetic RNA and DNA oligonucleotides used in this study were synthetized by Integrated DNA Technologies Inc. (IDT, Coralville, IA, USA), and their names associated with their sequences, as summarized in Table 1.

### 4.2. Splinted 5′ Ligation RT-qPCR Method

#### 4.2.1. Adapter Sequence

An RNA adapter was used to lengthen unusually short RNAs of interest (the doRNAs doRNA and C-doRNA) in their 5′ end. We selected an RNA adapter sequence that differed from the precursors of doRNAs and did not map to the human or mouse transcriptomes. We ascertained that adapter-ligated doRNA or C-doRNA sequences did not create a pre-existing RNA in human or mouse. Briefly, we used an online random generator (http://www.faculty.ucr.edu/~mmaduro/random.htm) (accessed on 3 August 2021) to generate various 10-nt sequences. Then, we selected sequences with the lowest capacity to bind to human or mouse transcripts. To that end, we reverse-complemented different potential adaptors and used BLAST nucleotide (blastn) searches from NCBI to verify if there were sequences in the human or mouse transcriptome that could correspond to the sequence of our adaptor. No significant similarity was found. Through blastn, we first searched for any correspondence with the “AATGGTTGTC” sequence (the reverse complement of our adapter) or the “CTGTTGGTAA” sequence (the complement of our adapter). Then, we selected the “Genomic + transcript databases” using the “Human genomic plus transcript (Human G + T)” or the “Mouse genomic plus transcript (Human G + T)” to verify in both organisms. The program used was MegaBLAST (optimized for highly similar sequences). 

We carried on the same analysis for the extended doRNA and C-doRNA, respectively “GACAACCTTGACTCTTAGCGG” and “GACAACCTTCGACTCTTAGCGG”, on NCBI. We did not find these sequences in the human database, while we found some matches in the mouse. However, the polyadenylation step during the reverse transcription, combined with targeted amplification, permitted to distinguish our extended doRNA or C-doRNA from them. Finally, we tested these sequences on miRBase (https://www.mirbase.org/cgi-bin/blast.pl) (accessed on 3 August 2021) and tRFdb (http://genome.bioch.virginia.edu/trfdb/search.php) (accessed on 3 August 2021) and found no similar sequence.

#### 4.2.2. Splint Sequences

This DNA sequence was inversely complementary to the adapter and to the RNAs to be detected in order to bring the two sequences together and favor their specific ligation, as schematized in Figure 1. Various splint sequences (Table 1) were designed so as to leave between 2 and 5 nt free at the 3′ end of the adapter and 2 nt free at the 5′ end of the RNAs to be detected, which was previously reported to facilitate ligation by the T4 RNA ligase [21].

#### 4.2.3. Adapter–Splint Annealing

The synthetic RNA adapter and DNA splint oligonucleotides were suspended in water to a final concentration of 100 μM. For the annealing step, the adapter (20 μM) was mixed with the splint (10 μM) in annealing buffer (200 mM Tris-HCl, 750 mM KCl, pH 7.5). Different annealing protocols were tested, as shown in Supplementary Table S1. The annealing protocol, kept in all experiments, consisted in heating at 65 °C for 3 min, followed by gradual cooling at a rate of 0.1 °C/s to reach a final temperature of 25 °C for 5 min. 

#### 4.2.4. Splinted 5′ Ligation RT-qPCR

The annealed adapter/splint was added to 300 ng of total RNAs and 18 µL of ligation master mix containing 10 units T4 RNA ligase (New England Biolabs, M0437M, Whitby, Ontario, Canada), 10% DMSO, 25% PEG 8000 (New England Biolabs, Whitby, Ontario, Canada), 1 mM ATP (New England Biolabs, M0437M, Whitby, Ontario, Canada) and 20 units SUPERase•In RNAse inhibitor (Thermo Fisher Scientific, AM2694, Waltham, MA, USA) in T4 RNA ligase reaction buffer (50 mM Tris-HCl, 10 mM MgCl_2_, 1 mM DTT, pH 7.5). The 23 µL of ligation was allowed to proceed for 1 h at room temperature (RT), after which a 1:1 volume of stop ligation buffer (1 M Tris-HCl, 0.1 M EDTA, pH 8.0) was added. This step lengthened doRNA and C-doRNA to 22 nt and 23 nt RNAs, respectively. Two (2) µL of total ligated RNA were used for RT using the miRCURY Locked Nucleic Acid (LNA)-modified microRNA PCR Assay (QIAGEN Inc., Toronto, ON, Canada) and the oligo-d(T) primer with 5′ universal tag included in the miRCURY LNA RT Kit (QIAGEN Inc., Toronto, ON, Canada; catalog number #339340). After cDNA 1/10 dilution, qPCR was performed using miRCURY LNA SYBR^®^ Green PCR Kits (QIAGEN Inc., Toronto, ON, Canada) in 96-well plates (Bio-Rad, Mississauga, ON, Canada) using the CFX96 Touch™ Real-Time PCR Detection System (Bio-Rad, Mississauga, ON, Canada) and specific Custom LNA Oligonucleotides for the doRNA (No 339317, ad3-d-621278, Catalog no YCP0054421, QIAGEN Inc., Toronto, ON, Canada) and C-doRNA (No 339317, ad3-C-d-621381_1, Catalog no YCP0054420, QIAGEN Inc., Toronto, ON, Canada) amplification. The qPCR temperature cycling protocol was as follows: denaturation step at 95 °C for 10 min, followed by 45 cycles of denaturation at 95 °C for 10 s and annealing/elongation at 60 °C for 1 min, with a ramp of 1.6 °C/s. Then, as melting curve was generated by heating from 70 °C to 90 °C with 0.5 °C increments, 10 sec dwell time, and plate read at each temperature. The RT-qPCR data were normalized either using endogenous small nucleolar RNA U6 or the RNA spike-in provided in the miRCURY LNA microRNA PCR Assay kit.

#### 4.2.5. Standard Curve

doRNA, C-doRNA, miR-25 and miR-30a copy numbers were determined using a standard curve established using the corresponding synthetic RNA oligonucleotides (IDT, Coralville, IA, USA) serially diluted 1/10th to obtain from 6.1 × 10^9^ to 6.2 × 10^2^ copies, covering a range of concentration of 7 logs. For each standard curve, the cycle quantitation (Cq) values with the corresponding copy numbers were plotted, and the linear curve equation, correlation coefficient (*R*^2^) and *p* value were calculated.

### 4.3. doRNA and C-doRNA Product Cloning and Sequencing 

The pBluescript II plasmid (Addgene, MA, USA) was linearized using EcoRV restriction enzyme (New England Biolabs, MA, USA), inducing a cut within the LacZ gene, followed by dephosphorylation of the blunt ends using Antarctic phosphatase enzyme (New England Biolabs, MA, USA) to prevent plasmid recircularization. In parallel, doRNA or C-doRNA products amplified by splinted 5′ ligation RT-qPCR were analyzed by 20% polyacrylamide, 8M urea gel electrophoresis, revealed by nucleic acid staining solution (RedSafe Dye TM, Montreal Biotech Inc, Montreal, QC, Canada) and the bands excised for subsequent gel extraction using QIAquick gel extraction (QIAGEN, Toronto, ON, Canada). The purified RNAs were phosphorylated using T4 polynucleotide kinase (New England Biolabs, MA, USA). The dephosphorylated digested plasmid was then mixed with the phosphorylated doRNA or C-doRNA insert (1:8, plasmid:insert molar ratio) in a ligation reaction catalyzed by T4 DNA ligase (New England Biolabs, MA, USA). The ligation products were transformed into bacteria, which were spread on LB plates containing 50µg/mL ampicillin and X-Gal (5-Bromo-4-chloro-3-indolyl-β-D-galactopyranoside, Sigma-Aldrich, Oakville, ON, Canada). Bacteria transformed with a plasmid not expressing LacZ gene (containing an insert) grew as white colonies and clones were selected and expanded for plasmid DNA purification. Inserts were sequenced using the reverse or forward T7 oligonucleotides at the SANGER Sequencing platform of the CHU de Québec-Université Laval Research Center (http://www.crchudequebec.ulaval.ca/en/services/sanger-and-sequenom/services/sanger-sequencing/) (accessed on 3 August 2021). All five clones derived from the band amplified with the doRNA primer contained the doRNA-extended DNA sequence (e.g., doRNA with the adaptor at one end, and the poly (dT) and universal sequence at the other end), whereas all five clones derived from the band amplified with the C-doRNA primer contained the C-doRNA-extended DNA sequence. These sequencing results supported the specificity of our splinted 5′ ligation RT-qPCR method for doRNA vs. C-doRNA detection.

### 4.4. Validation of the Splinted 5′ Ligation RT-qPCR Detection Method 

Six (6) samples, each containing one of the following synthetic oligonucleotides (2.5 × 10^7^ copy number) or water (used as control), and total RNA (300 ng) extracted from cultured N2a cells, were prepared in a final volume of 2 µL: doRNA, C-doRNA, mutated C-doRNA (single G2 to U2 substitution), 5′ extended C-doRNA (with 10 nt from the ITS1 sequence), 3′ extended C-doRNA (with 10 nt from the 5.8S rRNA). RT-qPCR data were normalized on small nuclear RNA (snRNA) U6 levels, and the fold change was calculated versus the control sample (water). Each sample was subjected to splinted 5′ ligation RT-qPCR detection of doRNA and C-doRNA. Cq data were considered only when the standard deviation for the technical duplicates was below 0.3. 

### 4.5. Primary and Cultured Human and Mouse Cells and Tissue

#### 4.5.1. Polymorphonuclear (PMN) Leukocyte Isolation

Venous blood was obtained from healthy volunteers for human PMN and total blood from healthy 12 to 15-week-old mice for mouse PMN, as described in Duchez et al. [31]. PMN derived from four healthy blood donors, or four mice were pooled.

#### 4.5.2. Platelet Isolation

Platelets were isolated from venous blood collected from healthy donors, as described in Laffont et al. [32]. Platelets derived from four healthy blood donors were pooled.

#### 4.5.3. Cell Lines

The human cell lines used in this study were human umbilical vein endothelial cells (HUVEC; Stem Cell Technologies, Vancouver, BC, Canada) and human embryonic kidney 293 (HEK293) cells (ATCC, Manassas, VA, USA). The mouse cell lines used in this study were neuronal N2a cells (neuro2A, ATCC, Manassas, VA, USA) and NIH/3T3 cells (ATCC, Manassas, VA, USA). Cells were cultured in their appropriate medium (Supplementary Table S1), supplemented with 10% (*v*/*v*) fetal bovine serum (FBS, System Biosciences), 1 mM sodium pyruvate, 100 units/mL penicillin, 100 μg/mL streptomycin and 2 mM L-glutamine in a humidified incubator under 5% CO_2_ at 37 °C.

#### 4.5.4. Mouse Tissue

A sample of brain cortex (old cerebellum 3; OC3) was collected from a two-year-old mouse, using a protocol approved by the Université Laval Animal Welfare Committee and in accordance with the guidelines, regulations and requirements of the Canadian Council of Animal Care for Animals Used for Scientific Purposes. 

### 4.6. Total RNA Isolation

Total RNA samples were obtained using TRIzol^®^ or TRIzol LS^®^ reagent (Invitrogen Life Technologies, Carlsbad, CA, USA) for liquid samples, and contaminated DNA was removed by treatment with DNase I (M0303S, New England Biolabs, MA, USA), following the manufacturer’s recommendations.

### 4.7. Validation of Small RNA-Sequencing (RNA-Seq) Data

The same total RNA samples, derived from the primary and cultured human and mouse cells and tissue mentioned above, were analyzed by small RNA-Seq in the ~8 to 30-nt window of RNA sizes. The RNA-Seq data are described in detail in the manuscript submitted as a separate Standard Paper and were provided through the standard analysis pipeline of Arraystar Inc. (Rockville, MD, USA; available on https://www.arraystar.com/) (accessed on 3 August 2021) and refined using R (Free Software Foundation). Only the reads that were completely identical in both lengths and sequences were assembled as a unique read. Small RNA read counts were normalized as reads per million (RPM) small RNA alignments. 

### 4.8. Statistical Analyses 

Statistical analyses were performed using Prism 7 (GraphPad Software, Inc., available at https://www.graphpad.com/; accessed on 3 August 2021). In vitro experiments were conducted in biological triplicates (minimum) with type alpha error set to 0.05 (5%). Statistical significance was determined by one or two-way ANOVA with Holm–Sidak’s post hoc test for multiple comparisons or t-test. Linear regression comparisons were verified by the Fisher test, so the *p*-value indicated the strength of the linear regression.

## Figures and Tables

**Table 1:** RNA and DNA oligonucleotides used in this study.

Name	Sequence *
doRNA	/5Phos/rGrArCrUrCrUrUrArGrCrGrG
C-doRNA	/5Phos/rCrGrArCrUrCrUrUrArGrCrGrG
adapter-doRNA	/5Phos/rGrArCrArArCrCrArUrUrGrArCrUrCrUrUrArGrCrGrG
adapter-C-doRNA	/5Phos/rGrArCrArArCrCrArUrUrCrGrArCrUrCrUrUrArGrCrGrG
adapter	/5Phos/rGrArCrArArCrCrArUrU
splint 2:2	CCGCTAAGAGTTGGTTGTC
splint 2:3	CCGCTAAGAGTGGTTGTC
splint 2:4	CCGCTAAGAGTGTTGTC
splint 2:5	CCGCTAAGAGTTTGTC
mutated C-doRNA	/5Phos/rCrUrArCrUrCrUrUrArGrCrGrG
5′ extended C-doRNA	/5Phos/rCrGrArCrCrUrCrGrUrArCrGrArCrUrCrUrUrArGrCrGrG
3′ extended C-doRNA	/5Phos/rCrGrArCrUrCrUrUrArGrCrGrGrUrGrGrArUrCrArCrUrC

* r, denotes a ribonucleotide. Unmarked nucleotides are deoxyribonucleotides. /5phos/, 5′ Phosphate modification. Underlined nucleotides denote mutations or extensions.

## Data Availability

doRNA and C-doRNA sequences were deposited to the DNA Data Bank of Japan (DDBJ; entry ID for doRNA: 5f876b84a3c882000c844366; entry ID for C-doRNA: 5f876b84a3c882000c844366; both to be released upon publication).

## Supplementary Materials

The following are available online at https://www.mdpi.com/article/10.3390/ncrna7030059/s1, Table S1: List of different annealing protocols tested, Figure S1: Optimization of the adapter:target ratio, Figure S2: Optimization of the splint:adapter ratio, Figure S3: Utilization of a pre-annealing step of the splint:adapter, Figure S4: Assessment of the importance of reaction medium components.
