# Supplementary material for: A New Specific and Sensitive RT-qPCR Method Based on Splinted 5′ Ligation for the Quantitative Detection of RNA Species Shorter than microRNAs
Source: Noncoding RNA. 2021 Sep 18;7(3):59. doi: 10.3390/ncrna7030059 (PMC8482087; doi:10.3390/ncrna7030059)
Supplement: Supplementary file 1 [file ncrna-07-00059-s001.zip › ncrna-1346777-supplementary.pdf]

Supplementary Table S1. List of different annealing protocols tested.

Cycle	Annealing protocol	Cq (doRNA)	Cq (C-doRNA)
A	3 min 65°C, decreasing of 0.1°C/sec until 25°C, 5 min 25°C	18.16	17.82
B	3 min 95°C, decreasing of 0.1°C/sec until 25°C, 5 min 25°C	22.24	21.74
C	1 min 65°C, decreasing of 0.1°C/sec until 25°C, 5 min 25°C	18.25	17.93
D	1 min 95°C, decreasing of 0.1°C/sec until 25°C, 5 min 25°C	21.62	21.24

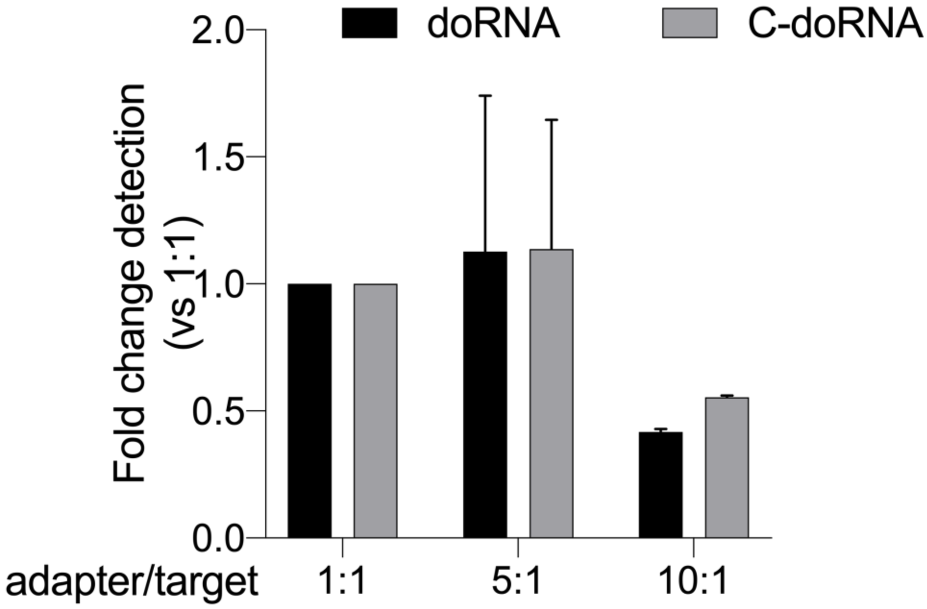

Supplementary Figure S1. Optimization of the adapter:target ratio. Various adapter:target ratios were assessed for their ability to optimize 5′ ligation RT-qPCR detection of doRNA and C-doRNA. Cycle quantitation (Cq) data were normalized on small nuclear RNA U6, and the fold changes were calculated versus the 1:1 adapter•target condition (mean ± SEM; n = 3 independent experiments). Two-way ANOVA with Holm Sidak's post-hoc test.

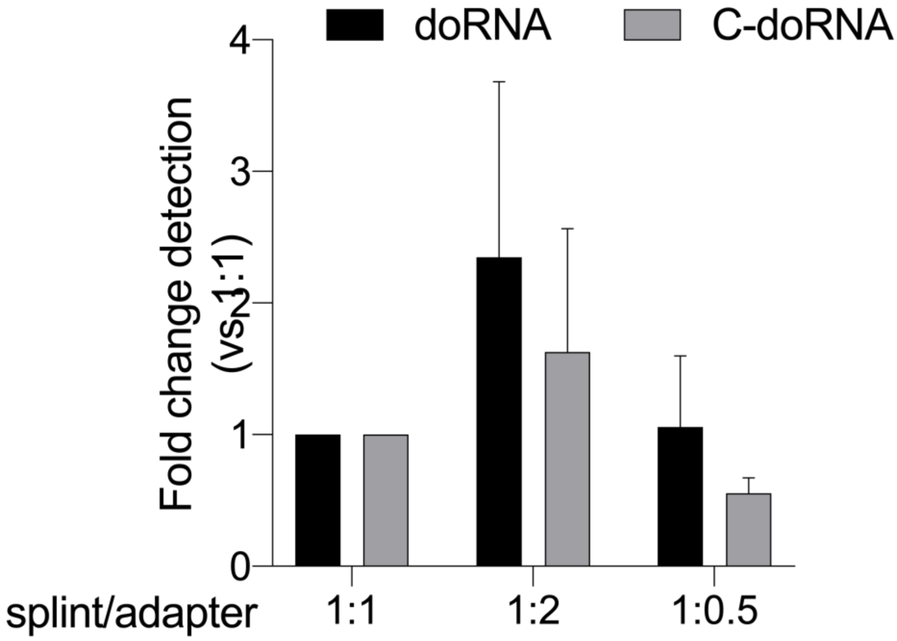

Supplementary Figure S2. Optimization of the splint:adapter ratio. Various splint:adapter ratios were assessed for their ability to optimize 5′ ligation RT-qPCR detection of doRNA and C-doRNA. Cycle quantitation (Cq) data were normalized on small nuclear RNA U6, and the fold changes were calculated versus the 1:1 splint:adapter condition (mean ± SEM; n = 4 independent experiments). Two-way ANOVA with Holm Sidak's post-hoc test. .

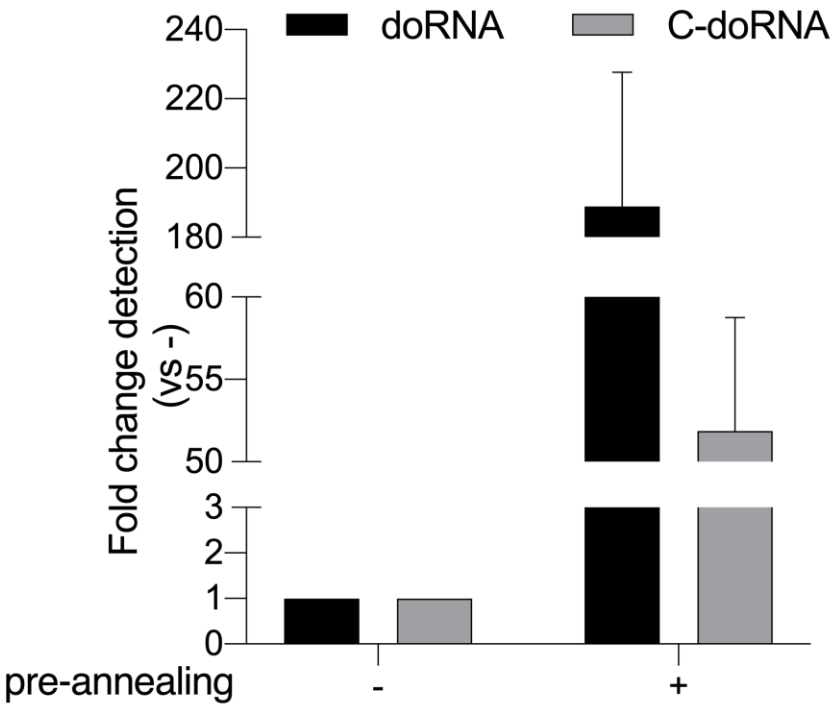

Supplementary Figure S3. Utilization of a pre-annealing step of the splint:adapter. We assessed whether pre-annealing the splint with the adapter optimized 5′ ligation RT-qPCR detection of doRNA and C-doRNA. Cycle quantitation (Cq) data were normalized on small nuclear RNA U6, and the fold changes were calculated versus the negative control (mean ± SEM; n = 2 independent experiments).

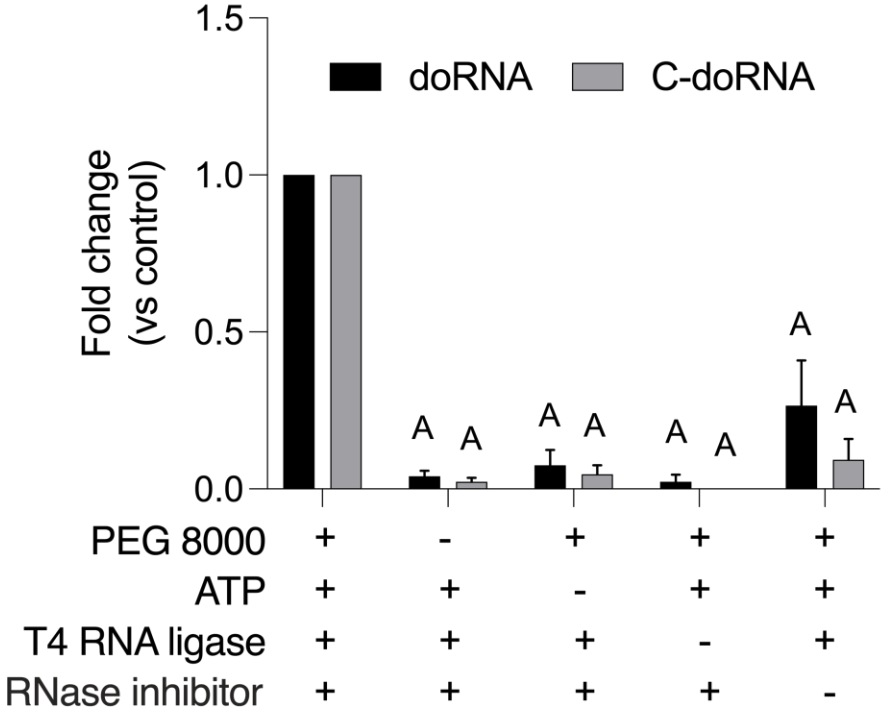

Supplementary Figure S4. Assessment of the importance of reaction medium components. The importance of the indicated, regular components of the splinted 5′ ligation buffer was assessed by quantitatively evaluating doRNA and C-doRNA detection in their absence. Cycle quantitation (Cq) data were normalized on U6 RNA, and the fold changes were calculated versus the optimized buffer condition (control) containing all the components (mean ± SEM; n = 3 independent experiments). The 4:2 splint was used in this experiment. Two-way ANOVA with Holm Sidak's post-hoc test. A, $p < 0.0001$.
